# Does *Trema micranthum* (L.) Blume Produce Cannabinoids?

**DOI:** 10.3390/plants13141951

**Published:** 2024-07-17

**Authors:** Gerlon de Almeida Ribeiro Oliveira, Omar Enrique Estrada-Semprun, Luciano Chaves Arantes, Patrícia Marques Rodrigues, Rebekah Alves Ribeiro, Christopher William Fagg, Pérola Oliveira Magalhães, Yris Maria Fonseca-Bazzo, Damaris Silveira

**Affiliations:** 1 Pharmacy Department, Health Sciences School, University of Brasília, Brasilia 70910-900, Brazil; gerlon.oliveira@unb.br (G.d.A.R.O.); pittieri@gmail.com (O.E.E.-S.); patriciamarques@unb.br (P.M.R.); rebekah.alves@aluno.unb.br (R.A.R.); perolam@hotmail.com (P.O.M.); yrisfonseca@hotmail.com (Y.M.F.-B.); 2 Laboratory of Forensic Chemistry and Physics, Institute of Criminalistics, Civil Police of the Federal District, Brasilia 70610-907, Brazil; lca1969@gmail.com; 3 Department of Botany, Institute of Biological Sciences, University of Brasília, Brasilia 70910-900, Brazil; acaciafagg@gmail.com

**Keywords:** Cannabaceae, phytocannabinoids, cannabidiol, FBBBS

## Abstract

There are inconclusive claims in the scientific literature that the species *Trema micranthum*, widely distributed throughout the Brazilian territory, may produce phytocannabinoids, potentially serving as an alternative to *Cannabis sativa*. In this study, we conducted a comprehensive investigation to assess the presence of phytocannabinoids in two *Trema micranthum* samples collected in the Midwest region of Brazil. In trying to detect cannabinoids in *T. micranthum*, a recommended cannabis screening test was employed, the Fast Blue BB Salt (FBBBS) colorimetric assay, followed by thin-layer chromatography (TLC) and instrumental techniques: high-performance liquid chromatography coupled to diode array detector (HPLC-DAD) and gas chromatography coupled to mass spectrometry (GC-MS). When employed without chloroform extraction, the FBBBS reagent yielded positive results for extracts from all parts of *T. micranthum* (leaves, branches, fruits, and inflorescences). However, these initial positive results from the FBBBS test, suggesting the presence of cannabinoids, were not corroborated by FBBBS followed by chloroform extraction, TLC, or the instrumental techniques used in this study. These additional outcomes suggest that the positive FBBBS test results were likely due to the presence of other phenolic compounds rather than phytocannabinoids. For example, the presence of vitexin-like compounds in *T. micranthum* extracts might explain the positive FBBBS test results. Therefore, new assertions that *T. micranthum* produces cannabinoids will require the support of more selective experiments to avoid false-positive claims based on less selective screening tests.

## 1. Introduction

Cannabinoids are aryl-meroterpenoid compounds that can originate from natural sources (plants), synthetic production, or endogenous processes. They exhibit activity at cannabinoid receptors, a key component of the endocannabinoid system [1]. In addition to these receptors, the endocannabinoid system includes endogenous cannabinoids (endocannabinoids) and the enzymes responsible for their degradation [2]. This system is widely distributed throughout the human organism and plays an important role in various physiological processes, including antinociception, learning and memory, neuroprotection, and the modulation of immunological processes, among others [3]. Due to its involvement in these critical functions, the endocannabinoid system has been proposed as a therapeutic target for the treatment of several health issues.

There is a growing global interest in cannabinoid derivatives (natural or synthetic) for medical purposes, particularly following the United Nations’ official recognition of the therapeutic value of cannabinoids in 2020 and the subsequent remotion of *Cannabis* from Schedule IV of the 1961 Single Convention on Narcotic Drugs [4,5].

The cannabinoid industry is experiencing rapid expansion. The global cannabinoid market was valued at USD 22.1 billion in 2021 and is projected to reach USD 154.2 billion in 2031, with a Compound Annual Growth Rate (CAGR) of 22.2%. This market is primarily segmented into cannabidiol (CBD), cannabinol (CBN), cannabigerol (CBG), and cannabidiolic acid (CBDA) [6].

The number of countries attempting to establish or have already implemented regulations permitting the medicinal use of cannabinoids (mainly CBD) is increasing year by year. However, the subject remains controversial. As a result, the search for alternative sources of phytocannabinoids other than *Cannabis sativa* is a strategy to provide the market with cannabinoids while avoiding the restrictions associated with the cultivation of *C. sativa*.

The Cannabaceae Martinov family is distributed in tropical and subtropical regions [7] and comprises nine genera [8]. One of them is the genus *Trema* Lour., which includes 20 species found in tropical and subtropical regions. While the *Trema* genus has been minimally studied from a chemical perspective, it has garnered interest within the research community due to reports suggesting the presence of cannabinoids [9,10,11,12]. Van Velzen and Schranz (2021) proposed that some cannabinoid biosynthesis genes originated from a common ancestor of *Cannabis* and related genera such as *Humulus*, *Parasponia,* and *Trema* [13]. Putative cannabinoid biosynthesis genes were also found in *Radula marginata* Gottsche, Lindenb. & Nees, a liverwort species from the bryophyte division of lower plants [14]. *Radula marginata* and *Radula perrottetii* Gottsche ex Steph. are known to produce many types of phytocannabinoids, including analogs of cannabigerolic acid (CBGA), Δ^9^-tetrahydrocannabinoic acid (THCA), and Δ^9^-THC bibenzyl. However, the cannabinoid oxidocyclase enzyme genes encoding THCA synthase (THCAS), CBDA synthase (CBDAS), and cannabichromenic acid synthase (CBCAS) are specific to *Cannabis sativa* L. [13].

Nonetheless, recent chemical analysis has revealed the presence of CBN, THC, and CBD in the chloroform fraction of the methanol extract from the inflorescences of nine specimens of *Trema orientalis* (L.) Blume, collected from different regions of Thailand. CBN was found to be the major component in all nine analyzed samples, while small amounts of THC were detected in four samples and CBD in two samples [11].

**Figure 1:**
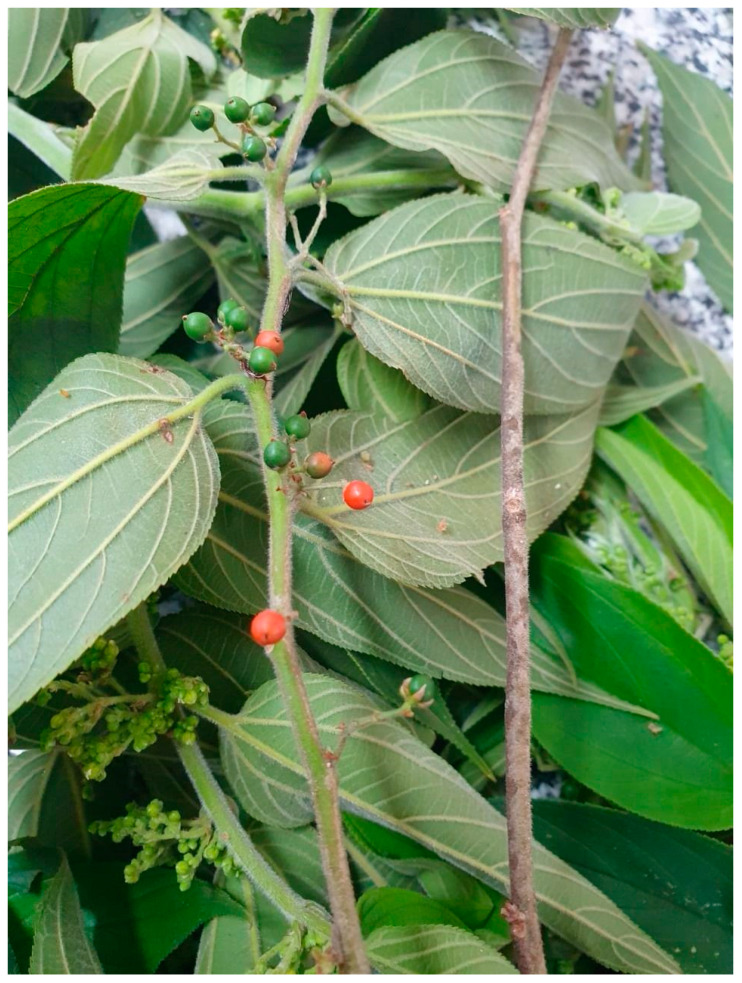
Leaves, inflorescences, unripe and ripe fruits, and branches from one of the *Trema micranthum* (L.) Blume specimens included in this work.

According to the Reflora Project, two *Trema* species are native to Brazil: *Trema micranthum* (L.) Blume and *Trema molle* (Willd.) Blume [15]. *Trema micranthum* (Figure 1) occurs in all Brazilian regions [15] and also throughout the Neotropics up to Florida in the USA. In Brazil, *T. micranthum* is a pioneer tree that grows in rainforests, gallery forests, and semi-deciduous forests. It is a morphologically variable species [16]. The leaves and bark are utilized in traditional medicine for their wound-healing, anti-rheumatism, and anti-syphilis properties [17,18]. Additionally, a decoction of leaves is employed in Brazilian folk medicine as an antidiabetic remedy, with its hypoglycemic activity confirmed by in vivo assays in alloxan-induced diabetic rats [19]. However, despite this species’ use for medicinal purposes and as fodder for cattle and small animals [18], there are reports of its neurotoxic and hepatotoxic effects [20,21,22,23,24,25,26,27,28]. Wouters et al. (2013) reported that 20 g/kg of *T. micranthum* leaves led to tachypnea, severe dyspnea, cyanotic mucous membranes, and other clinical toxicity signals in sheep [29].

In 2023, the Brazilian media widely publicized the discovery of cannabidiol in *T. micranthum* inflorescences and fruits; however, no scientific publication corroborating this finding has been identified, except for a report of positive results for Fast Blue B and Duquenois-Levine reagents in a histochemical analysis of *T. micranthum* trichomes [12].

As for other *Trema* species, few studies on the chemical composition of *T. micranthum* are available in the literature [19]. Several compounds were isolated from an ethanol extract from leaves and branches, including paprazine, ursolic acid, corosolic acid, β-sitosterol, 3β-O-β-D-glucopyranosyl sitosterol, and vitexin [30]. A chemical profile by HPLC-ESI-MS^2^ of an ethanol extract from *T. micranthum* leaves collected in the northern region of Brazil showed the presence of C-glycosides of the flavonoids orientin, isoorientin, vitexin, and isovitexin [31].

Given the limited knowledge regarding the chemical composition of *T. micranthum* and the global interest in alternative sources of phytocannabinoids, the potential presence of cannabinoids among this species’ secondary metabolites was investigated.

## 2. Results

### 2.1. Colorimetric Assay (Fast Blue BB Salt Reagent) 

One of the most common methods to identify potential *Cannabis* samples is through colorimetric assays. Fast Blue BB Salt (FBBBS) reagent is widely used as a preliminary test to determine the presence of cannabinoids in a sample, mainly Δ^9^-THC, CBN, and CBD. There are different ways to perform this colorimetric test: directly on filter paper, in the extractive solutions, or in an aqueous-chloroform biphasic solution. The latter is preferred due to its improved specificity to phytocannabinoids. The presence of cannabinoids is inferred by the appearance of a reddish color or, in the biphasic test, when the color of the lower (chloroform) phase changes to a reddish color [32,33]. To avoid false-positive assays, the biphasic FBBBS test is used in the Civil Police of the Federal District of Brazil to detect cannabinoids for forensics purposes. This test helps to differentiate specific reactions from nonspecific ones, as a reddish upper aqueous phase can sometimes appear due to nonspecific reactions. Nonetheless, colorimetric tests continue to be only screening tests, and their results must be confirmed with more specific analytical techniques such as chromatography.

**Figure 2:**
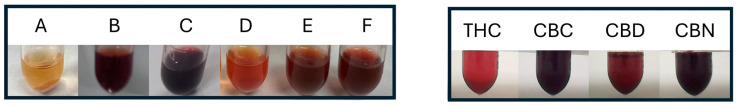
Colorimetric assay results using Fast Blue BB Salt (FBBBS). Reaction blank (**A**), and methanol extracts from *Trema micranthum* branches (**B**), leaves (**C**), ripe fruits (**D**), unripe fruits (**E**), and inflorescences (**F**), in comparison with the analytical standards of delta-9-tetrahydrocannabinol (THC), cannabichromene (CBC), cannabidiol (CBD), and cannabinol (CBN). Photos were taken after adding the methanol extracts to FBBBS, showing color changes indicative of phytocannabinoids.

Figure 2 shows the results of *T. micranthum* extracts from branches (B), leaves (C), ripe fruits (D), unripe fruits (E), and inflorescences (F), compared with THC, CBC, CBD, and CBN analytical standards, as well as a reaction blank used as a negative control (A). The colorimetric test results suggested the presence of cannabinoids in the methanol extracts of *T. micranthum*.

**Figure 3:**
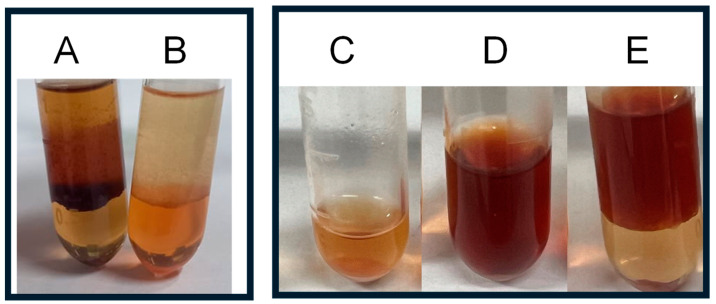
FBBBS colorimetric assay in biphasic mode for *Trema micranthum* leaves (**A**) and cannabidiol (**B**), and results of this test applied to the analytical standard vitexin (in methanol), step by step (**C**–**E**): (**C**) addition of vitexin solution to FBBBS, (**D**) addition of 0.1 mol/L NaOH and mixing, and (**E**) addition of chloroform and mixing.

As further demonstrated in the HPLC-DAD results (Section 2.3), vitexin-like substances were found in *T. micranthum* samples. Thus, the possibility of these flavonoids causing false-positive colorimetric assays was investigated. Figure 3 shows a stepwise colorimetric assay for the vitexin (analytical standard). Adding the methanol solution of vitexin to the FBBBS did not elicit a color change in the extract (Figure 3A). However, the solution turned bright red after adding 0.1 mol/L NaOH (Figure 3B). After the addition of chloroform to the solution, the chloroform (lower) phase regained a color like the reaction blank (Figure 2A), indicating a negative result for the presence of phytocannabinoids. The same behavior was observed for *T. micranthum* samples: the extractive solutions turned reddish upon the addition of FBBBS and NaOH solution (Figure 2B–F), but the substances that turned the most reddish remained in the aqueous phase when subjected to chloroform extraction (Figure 3A), similarly to how it occurs with vitexin (Figure 3C–E). On the other hand, cannabinoids migrate to the organic phase (Figure 3B). 

### 2.2. Thin Layer Chromatography (TLC)

**Figure 4:**
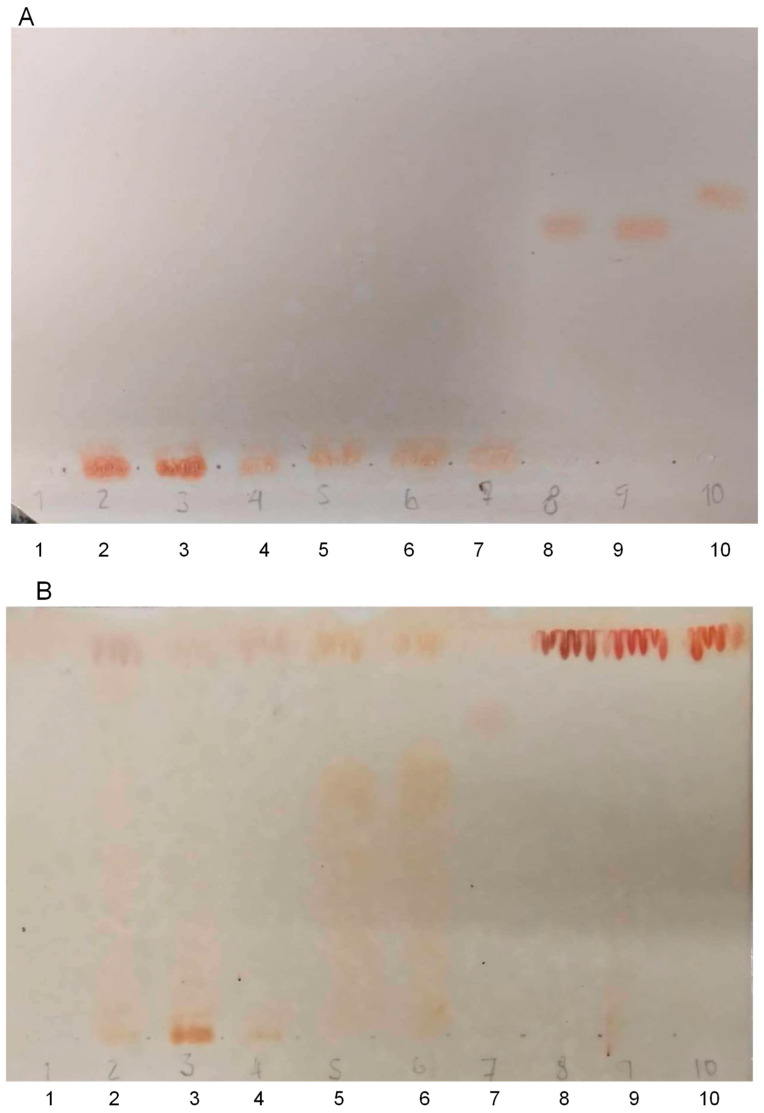
TLC analyses of methanol extracts from *Trema micranthum* and methanol solutions of analytical standards of cannabinoids and vitexin, using toluene (**A**) or ethyl acetate—acetic acid—formic acid—water (100:11:11:26, *v*/*v*) (**B**) as mobile phase. A mixture of the analytical standards CBN, THC, and CBD at 10 µg/mL (1), leaves (2), branches (3), inflorescences (4), unripe fruits (5), and ripe fruits (6) extracts of *T. micranthum*; analytical standards at 1000 µg/mL of vitexin (7), CBN (8), THC (9), and CBD (10). Detection reagent: Fast Blue BB Salt.

Considering the concerns about the specificity of Fast Blue BB Salt reagent in the detection of cannabinoids, *T. micrathum* extracts were analyzed by TLC in comparison with the main cannabinoids and with vitexin. The TLC analysis was conducted using two different conditions. The first condition (Figure 4A) had toluene as the eluent, as this is a routine method for cannabinoid detection in *C. sativa* [34]. In the second condition (Figure 4B), the mobile phase was a mixture of ethyl acetate, acetic acid, formic acid, and water (100:11:11:26, *v*/*v*) [35]. This method is appropriate for more polar compounds, such as flavonoids. Although some colors have developed in *T. micranthum* samples (as occurred in the colorimetric test), the spots corresponding to the extracts did not coincide with those of the cannabinoids.

Conversely, some of these spots had retention factors close to vitexin (Figure 4B, especially extracts from *T. micranthum* fruits 5 and 6). Vitexin also developed color in both conditions (Figure 4A,B, spot 7), but in the method for cannabinoids (Figure 4A), this substance did not elute since it is much more polar than cannabinoids. The samples of *T. micranthum* also did not present spots in the retention factor of vitexin. 

It is worth noting that cannabinoids were not observed by TLC in the standards prepared in a very low amount of standards (10 μg/mL) (Figure 4A, spot 1). The compounds were only observed in the standard solution (1000 µg/mL) (Figure 4A, spots 8 to 10). Thus, we have analyzed the samples using HPLC and GC-MS, which are more sensitive techniques.

### 2.3. High-Performance Liquid Chromatography Coupled to Diode Array Detector (HPLC-DAD) 

**Figure 5:**
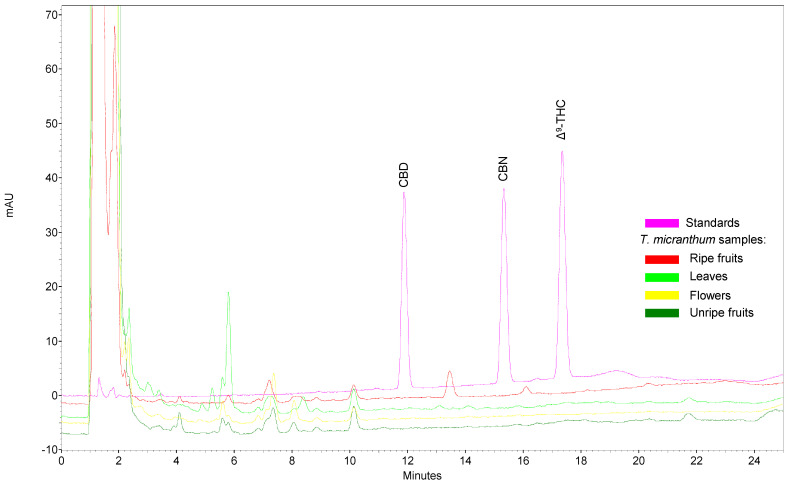
Chromatographic profile from *Trema micranthum* extracts and the main cannabinoids from *Cannabis sativa.* Pink line: analytical standards of CBD (~20 µg/mL), CBN (~10 µg/mL), and Δ^9^-THC (~30 µg/mL). Dark green line: *T. micranthum* methanol extracts of unripe fruits; yellow line: *T. micranthum* methanol extracts of inflorescences; light green line: *T*. *micranthum* methanol extracts of leaves; red line: *T. micranthum* methanol extracts of ripe fruits.

Figure 5 demonstrates the comparison between samples of *T. micranthum* and three cannabinoids from *C. sativa* (CBD, CBN, and Δ^9^-THC). None of the three cannabinoids were detected in methanol extracts from different parts of *T. micranthum*. This further confirms the absence of these cannabinoids in *T. micranthum* samples.

**Figure 6:**
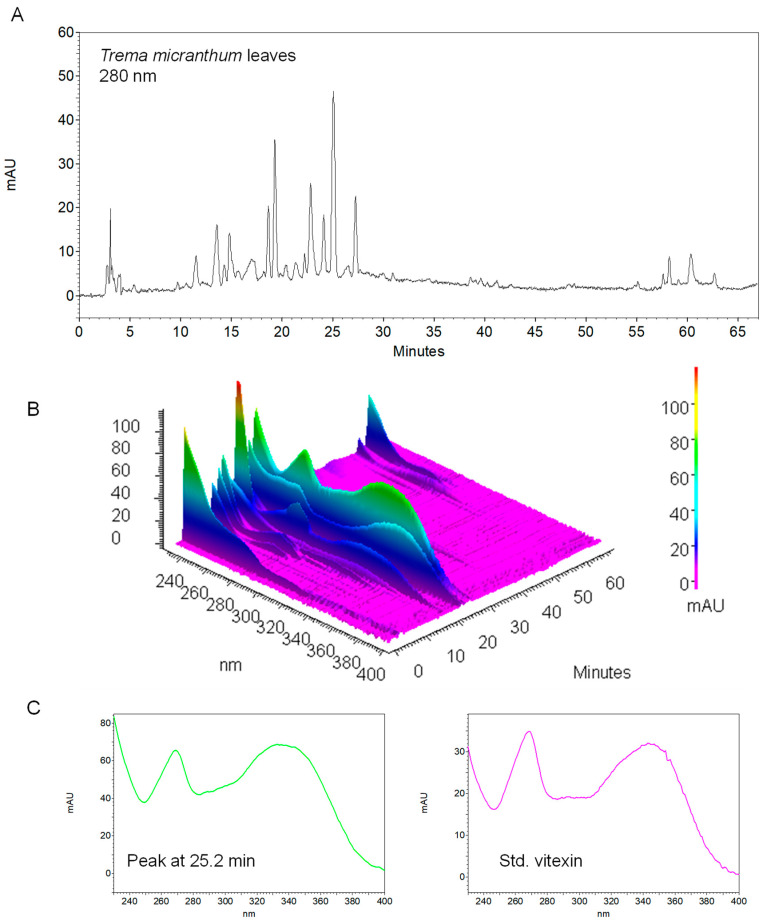
HPLC-DAD data using polyphenols method. (**A**,**B**) Chromatogram of methanol extracts of *Trema micranthum* leaves (at 280 nm in (**A**), and from 230 to 400 nm in (**B**)); (**C**) UV spectrum of the major peak in the chromatogram of *T. micranthum* leaves, at 25.2 min (**left**) and of the analytical standard of vitexin (**right**).

Since cannabinoids are less polar than most secondary metabolites, the chromatograms obtained through the initial method (Figure 5) showed the majority of compounds eluting very quickly, appearing together in the three initial minutes of the run. Then, a second method was employed to achieve a more informative chromatogram for polyphenols. This method began with a less strong mobile phase, allowing for better separation of peaks (chromatogram shown in Figure 6A). Some of these peaks exhibited absorption peaks characteristic of flavonoids (Figure 6B and left spectrum of Figure 6C), with absorption maxima in the range of 240 to 285 nm and 300 to 550 nm [36]. Specifically, certain peaks displayed spectra resembling those of vitexin derivatives (as observed by comparing the spectrum of a sample peak and the vitexin analytical standard in Figure 6C). It is important to note that the presence of vitexin derivatives in *T. micranthum* was already reported [31], although future research is necessary to confirm this hypothesis since the TLC band of vitexin (Figure 4B, spot 7) was not found in *T. micranthum* samples (Figure 4B, spots 2–6). 

### 2.4. Gas Chromatography Coupled to Mass Spectrometry (GC-MS)

**Figure 7:**
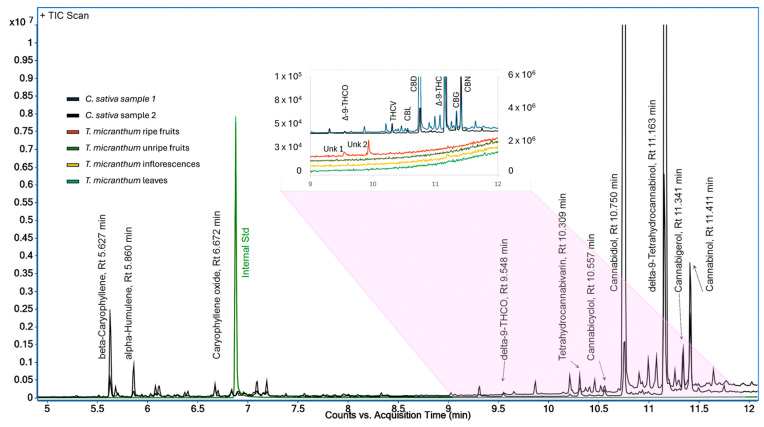
Total ion chromatograms (TIC) of *Cannabis sativa* and *Trema micranthum* samples, including leaves, inflorescences, unripe fruits, and ripe fruits. Some compounds detected in the *Cannabis sativa* samples are labeled: Delta-9-Tetrahydrocannabiorcol (Δ^9^-THCO); tetrahydrocannabivarin (THCV); cannabicyclol (CBL); cannabidiol (CBD); Delta-9-Tetrahydrocannabinol (Δ^9^-THC); cannabigerol (CBG); cannabinol (CBN). Unknown compounds from *T. micranthum*: Unk 1 and Unk 2.

Samples of *T. micranthum* and *C. sativa* were also analyzed by GC-MS. As expected, some cannabinoids were identified in the *C. sativa* samples by mass spectra comparison: cannabidiol (CBD), Δ^9^-THC, cannabigerol (CBG), and cannabinol (CBN) (Figure 7). Sesquiterpenes and the other phytocannabinoids were tentatively identified by comparing their mass spectra to those in the forensic mass spectral libraries from the Scientific Working Group for the Analysis of Seized Drugs (SWGDRUG MS Library version 3.13) and Cayman Chemical (Cayman Spectral Library v30052024) (Figure 7). 

**Figure 8:**
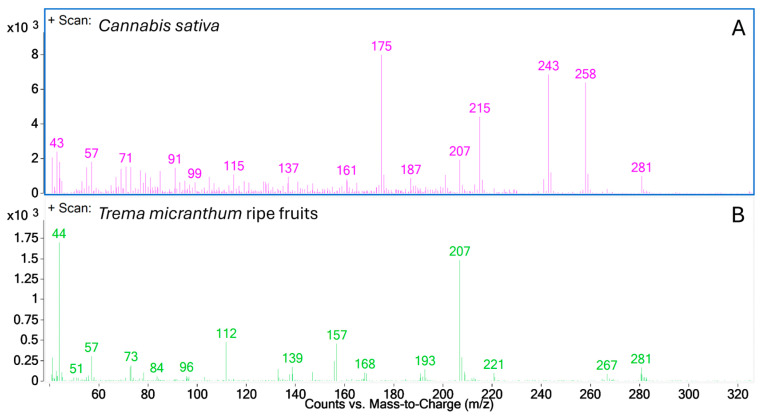
Mass spectra of peaks from *Cannabis sativa* (**A**) and *Trema micranthum* ripe fruit (**B**) samples at retention time 9.548 min.

Phytocannabinoids detected in the *C. sativa* samples appeared in the retention time range between 9 and 12 min. However, none of the *T. micranthum* samples exhibited peaks at the same retention times, except for ripe fruits, which showed two peaks. One of these peaks coincided with the retention time as Δ^9^-THCO and another at 9.932 min (Figure 7, inset). Despite the similar retention time of unknown peak 1 from the *T. micranthum* ripe fruit sample and Δ^9^-THCO from *C. sativa* sample, their mass spectra had different base peaks, and the Δ^9^-THCO molecular ion (*m*/*z* 258) was only present in the *C. sativa* sample mass spectrum (Figure 8).

**Figure 9:**
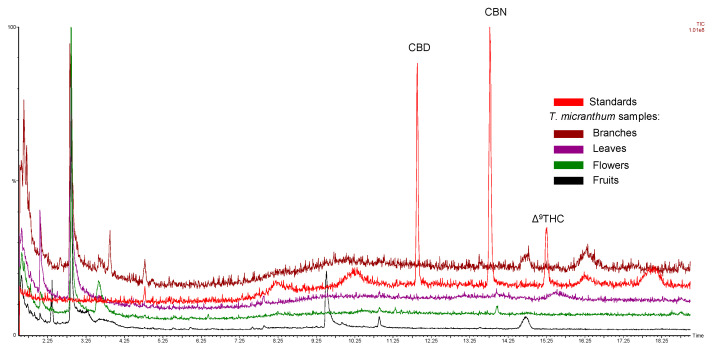
Total ion chromatograms (TIC) of analytical standards of CBD, CBN, and Δ^9^-THC and *Trema micranthum* samples, including branches, leaves, inflorescences, and fruits. *T. micranthum* samples did not present peaks with retention times or MS spectra of any cannabinoid.

To corroborate our observations, a GC-MS analysis of methanol extracts obtained from a secondary *T. micranthum* specimen, sourced from Jaraguá, GO, Brazil, yielded analogous results: cannabinoids were absent across all surveyed plant parts encompassing leaves, inflorescences, fruits, and branches (Figure 9).

## 3. Discussion

In the present investigation, the detection of cannabinoids within diverse methanol extracts derived from various parts of the *T. micranthum* plant was undertaken. While colorimetric assays suggested the presence of cannabinoids in the extracts of *T. micranthum*, such assertions were not corroborated by modified colorimetric procedure, TLC, HPLC-DAD, and GC-MS analysis findings. 

Through the examination of the separation pattern of the distinct extracts via TLC employing two different mobile phases and subsequent comparison of them with analytical standards of cannabinoids, the discernible bands attributable to the compounds present in the *T. micranthum* extracts did not align with those of the *C. sativa* samples. In the method for cannabinoids (Figure 4A), the spots from *T. micranthum* (2–6) and vitexin (7) developed reddish colors, similar to the spots of cannabinoids (8–10). However, the retention factors did not match. Thus, the reddish coloration of certain bands when subjected to FBBBS revelation does not decisively indicate the presence of cannabinoid-type compounds. This nuanced reddish color could be due to the presence of phenol-type compounds, which similarly react with the FBBBS reagent [32,37]. Overall, TLC results suggested the absence of cannabinoids in the analyzed samples. 

The HPLC-DAD analyses revealed that the compounds responsible for yielding positive screening tests for the presence of flavonoids in *T. micranthum* could be vitexin-like substances, as they exhibit similar UV spectra and vitexin also produces reddish solutions in colorimetric FBS tests. Flavonoids, including vitexin, are generally more polar compounds compared to cannabinoids. This difference in polarity allowed them to be differentiated from cannabinoids in chromatographic tests or, more simply, use liquid–liquid extractions. Cannabinoids are partitioned into the chloroform layer, while flavonoids are partitioned into the aqueous layer during liquid–liquid extraction with a water-immiscible solvent like chloroform.

Upon subjecting the *T. micranthum* extracts to high-performance liquid chromatography under conditions delineated in extant literature for cannabinoid detection and separation [38], it was observed that the components present in the extracts exhibited retention times markedly disparate from those of CBD, CBN, and Δ^9^-THC (Figure 5). It merits emphasis that the cannabinoid concentrations in the standard solution were 10–30 µg/mL, which presented peaks with high intensities. Consequently, if peaks with intensities equivalent to 10% of the least concentrated standard were present in the samples’ chromatograms, they would have been readily detected. In other words, even using a technique with a limit of detection as low as around 1 µg/mL, cannabinoids were not detected in *T. micranthum*. Considering that samples were prepared in a 1:20 drug-to-solvent ratio (DSR), each milliliter of solvent was used in the extraction of 50 mg of the plant material and, consequently, a limit of detection of 1 µg per mL of solvent is equivalent to 20 µg per gram of sample, or 20 mg/kg.

Furthermore, analyses were also carried out by GC-MS under conditions reported in the literature for the forensic detection of cannabinoid-type compounds [39]. The outcomes revealed that the retention times or the mass spectra of *T. micranthum* samples do not match those of cannabinoid compounds observed in the *C. sativa* samples (Figure 7) or analytical standards (Figure 9). There was only one peak with a similar retention time in the two plants, but while in the *C. sativa* sample, this peak was tentatively identified as Δ^9^-THCO (delta-9-tetrahydrocannabiorcol) by mass spectra similarity; in the *T. micranthum* ripe fruit sample, the peak presented fragment ions similar to those expected to dimethylpolysiloxane-related contamination (ions 73, 207, 221, 281), probably due to septum and/or column bleed. Other peaks did not present an MS library match with cannabinoids when compared to the Cayman spectral library v30052024 and the SWGDRUG MS Library version 3.13.

Napiroon et al. (2021) found cannabinoids while studying *Trema orientalis* (L.) Blume, a species closely related to *T. micranthum* [11]. While the authors compared retention times of analytical standards and sample peaks, mass spectral data—which could further confirm the identity of the compounds—were not included in the comparison. As is well-known and noted in the present work, different compounds may present peaks with the same retention time in any chromatographic technique. Mass spectra provide a second layer of security in confirming the identity of the substances that give rise to these peaks. Nevertheless, even if the potential methodological limitation of their study is set aside, the concentration of cannabinoids in the samples they analyzed exhibited significant variation: THC ranged from not detected to 90 mg/kg; CBD from not detected to 5 mg/kg; and CBN from 50 to 357 mg/kg. Considering this significant heterogeneity observed in the cannabinoid composition of *T. orientalis*, it would be possible to expect that some samples of *T. orientalis* or samples from other *Trema* species could produce less than around 20 mg/kg of any cannabinoid. This concentration is the approximate limit of detection obtained for HPLC-DAD in the present study.

## 4. Materials and Methods

### 4.1. Plant Material and Preparation of Extracts 

Access to the studied species, *Trema micranthum* (L.) Blume (Cannabaceae), was registered in the Brazilian National System of Management of Genetic Heritage and Associated Traditional Knowledge (SISGEN) under nº AAA6F36. The botanist, Dr. Christopher William Fagg, collected and identified the plant material in October 2023. A voucher (Fagg CW 2568) was deposited at the Herbarium of the University of Brasília (UB).

Samples of *T. micranthum* were collected in the Paranoá region, Brasília-DF, in the Brazilian Midwest, specifically in the Cerrado biome. Fruits, inflorescences, branches, and leaves were separated and dried in the shade in an oven with air circulation at 40 °C. One gram of different parts of *T. micranthum* (fruits—unripe and ripe, inflorescences, and leaves) was ground into a fine powder using a knife mill and subsequently soaked overnight with 20.0 mL of methanol (MeOH PA Tedia). Afterward, an ultrasound-assisted maceration (Unique-UltraSonic Cleaner, model Usc-2800A, with 220 W potency and 40 KHz frequency) was performed for one hour. After filtration, the obtained extractive solution [drug-to-solvent ratio (DSR) 1:20] was used for subsequent analyses. 

In a final attempt to identify cannabinoids in *T. micranthum*, another sample was collected in the Jaraguá municipality in the Goiás state, situated approximately 150 km away from the first site of collection. This tree did not present unripe fruits; thus, the inflorescences, ripe fruits, branches, and leaves were prepared as the other sample and underwent GC-MS analyses. The extracts from this secondary sample were compared to the three main cannabinoids (CBD, CBN, and Δ^9^-THC) prepared as described in the HPLC-DAD method (Section 4.4). Furthermore, the chromatographic peaks were compared to the NIST 11.0 databank.

Samples from two different seizures, comprising dried and pressed *C. sativa* aerial parts, were kindly supplied by the Institute of Criminalistics of the Civil Police of the Federal District of Brazil. All the experiments involving these samples were performed in the Chemistry and Physics Forensic Laboratory of the Institute of Criminalistics of the Civil Police of the Federal District of Brazil. Samples were ground into a fine powder and then extracted with MeOH for gas chromatography–mass spectrometry (GC-MS) analyses. An aliquot of 100 mg of the ground plant material was extracted with 1 mL of MeOH, homogenized by vortexing for 1 min, and further extracted using water-bath sonication for 10 min. Ten microliters of the extract were diluted in 1 mL of MeOH for GC-MS analyses. 

### 4.2. Colorimetric Assay

A colorimetric assay was conducted in a 2 mL plastic microtube using a method developed by Faubert Maunder (1969) with slight modifications [33,40]. The main modification was the substitution of 3,3′-dimethoxy-4,4′-biphenylbis(diazonium) zinc chloride (1:1:4) [CAS number 14263-94-6], also known as Fast Blue B Salt (FBBS), with 4-(benzoylamino)-2,5-diethoxybenzenediazonium chloride dichlorozinc (2:2:1) [CAS number 5486-84-0], known as Fast Blue BB Salt (FBBBS) [41].

Approximately 200 µL of methanol extracts from different parts of *T. micranthum* were mixed with 300 µL of methanol and 9 mg of a FBBBS preparation (1:100 dilution of FBBBS in anhydrous sodium sulfate). After 1 min of vortex-assisted homogenization, 0.5 mL of 0.1 mol/L NaOH aqueous solution was added. Then, 500 µL of chloroform was added, the mixture was vortexed, and the tubes were set aside to allow phase separation. The appearance of a reddish color after adding NaOH solution is a common, yet unreliable, indicator of cannabinoids in samples. Extracting the colored compounds into the chloroform layer (bottom) improves this method but is often overlooked in forensic practice. This paper explores the application of both methods for FBBBS screening: the initial colorimetric test with NaOH solution and the subsequent extraction with chloroform.

Cannabinoid standards and the flavonoid vitexin were also tested. The analytical standards THC, CBD, and CBN were purchased from Cayman Chemical, and vitexin Supelco was obtained from Merck. Ten microliters of each standard solution at 0.1 mg/mL in methanol were mixed with 490 µL of methanol in the microcentrifuge tubes with FBBBS, and the tests were performed as described for the *T. micranthum* samples.

### 4.3. Thin Layer Chromatography (TLC)

A straightforward TLC protocol for the identification of *C. sativa* constituents was conducted on TLC aluminum sheets coated with a 0.02 mm layer of silica gel 60G F254 (Merck). Elution was carried out employing two distinct mobile phases: (i) toluene; and (ii) a mixture of ethyl acetate, acetic acid, formic acid, and water (100:11:11:26, *v*/*v*) [35]. Samples analyzed: methanol extracts from *T. micranthum* and methanol solutions of analytical standards of cannabinoids (CBD, THC, and CBN) and vitexin. Vitexin and the cannabinoids were analyzed at 1000 µg/mL; additionally, a mixture of the cannabinoids at 10 µg/mL each was also analyzed. Spots were visualized after development with an aqueous solution (2 mg/mL) of Fast Blue B salt spray, adapted from Faubert Maunder [40,41]. 

### 4.4. High-Performance Liquid Chromatography Analysis Coupled with Diode Array Detector (HPLC-DAD)

HPLC-DAD analyses performed for cannabinoids detection were carried out in a Hitachi chromatograph with a DAD detector and quaternary gradient pump, following the German Pharmacopoeia (DAB 2018) conditions as described in the Knauer application note [38], with the following modifications: a C18 Merck column (Purospher RP-18 end-capped), with 25 cm × 4.6 mm × 5 µm dimensions, equipped with a guard column of the same stationary phase, heated at 40 °C; the mobile phase consisted of a gradient of acetonitrile, in one line, and an aqueous solution containing 8.6 g/L of phosphoric acid in another line; the runs were performed with acetonitrile varying from 64% to 82% in the first 16 min; remaining in this condition for 4 min; then taking two minutes to return to the initial level and remaining there for three minutes. The DAD detector operated in the range from 200 to 800 nm, while chromatograms at 225 and 306 nm were continuously monitored. The injection volume was 10 µL. Before the analysis, the *T. micranthum* extracts (DSR 1:20) were filtered through a 0.45 µm syringe filter.

Cannabidiol (CBD), cannabinol (CBN), and Delta-9-tetrahydrocannabidiol (Δ^9^-THC) (DRE-A10946000ME, DRE-A10946200ME, and DRE-A17405100ME, respectively), all from DR-Ehrenstoffer, were prepared for a final concentration of 10 µg/mL. For that, 10 µL of each standard, bought at 1000 µg/mL, were mixed with 970 µL of methanol in an HPLC vial. To confirm the retention times of each cannabinoid in the chromatogram, after the first injection, an additional 10 µL of CBD and 20 µL of Δ^9^-THC was added to the vial, resulting in a final solution of roughly 20 µg/mL of CBD, 10 µg/mL of CBN, and 30 µg/mL of Δ^9^-THC (more precisely, 19.5 µg/mL of CBD, 9.71 µg/mL of CBN, and 29.3 µg/mL of Δ^9^-THC). The HPLC and GC-MS chromatograms reported herein are from this second standard solution.

Another HPLC-DAD method was applied for screening polyphenolic substances, adapted from previous work [42]. The same equipment and column were used but at 25 °C. The mobile phase consisted of a gradient of 1% phosphoric acid solution and acetonitrile at 0.6 mL/min. The percentage of acetonitrile was as follows: 10% at the beginning, increasing to 30% at 40 min, and further to 50% by 50 min. It returned to the initial ratio by 51 min and remained there for the next 16 min. DAD operated from 230 to 400 nm, and the wavelengths of 280 nm and 354 nm were continuously extracted. Then, 10 µL was injected. The spectra of the main peaks were compared to an in-house spectral library of polyphenols built based on the same chromatographic method.

### 4.5. Gas Chromatography Coupled to Mass Spectrometry (CG-MS)

CG-MS analyses was carried out in two systems. In the first, developed in the Civil Police of Federal District Laboratory and previously reported [43], an Agilent 7890A gas chromatograph hyphenated to a 5975C mass spectrometer (GC–MS) (Agilent Technologies, Santa Clara, CA, USA) was used. Agilent MSD ChemStation and Enhanced ChemStation Data Analysis software (version E.02.02.1431) were used for GC–MS control and data visualization, respectively. The Automated Mass Spectral Deconvolution and Identification System (AMDIS; version 2.73) and NIST Mass Spectral Search Program (version 2.3) were both used for data analysis and compound identification. A 30 m chromatographic column (J&W DB-1ms Ultra Inert analytical column; 0.25 mm I.D., 0.25 µm film thickness, Agilent, part number 122–0132UI) was submitted to a temperature-programmed method consisting of 1 min at 100 °C; ramp at 20 °C/min up to 312 °C; temperature which was kept for 4.3 min, thus performing a total run time of 15.9 min. Injector, transfer line, MS source, and MS quadrupole temperatures were 280, 280, 300, and 150 °C, respectively. One microliter (µL) of each extractive solution from the *T. micranthum* samples was injected separately. Additionally, two extractive solutions from *C. sativa* samples were injected. Helium was the carrier gas at a flow rate of 1 mL/min, with a split ratio 1:20. Chromatograms were collected 1.8 min after injection, and a mass range from 40 to 450 *m*/*z* was scanned. Electron impact was set at 70 eV.

The sample collected in the municipality of Jaraguá, in turn, was analyzed in another chromatographic system (Clarus 680 GC, Perkin Elmer, Singapore) equipped with an Rtx 5 MS capillary column (30 m × 0.32 mm × 0.25 µm). The column was heated at 200 °C for 5 min, then the temperature increased from 10 °C/min to 240 °C; holding it there for 10 min. 1.5 microliters was injected. This chromatograph is coupled to a quadrupole mass spectrometer (Clarus SQ8 MS, Perkin Elmer, Singapore) as a detector, operating at 70 eV. Injector, transfer line, MS source, and MS quadrupole temperatures were 280, 280, 220, and 150 °C, respectively. A mass range from 35 to 500 *m*/*z* was collected in the chromatograms 1.5 min after injection. Cannabidiol (CBD), cannabinol (CBN), and Delta-9-tetrahydrocannabidiol (Δ^9^-THC) (DRE-A10946000ME, DRE-A10946200ME, and DRE-A17405100ME, respectively), all from DR-Ehrenstoffer, were used as cannabinoids reference.

## 5. Conclusions

Under the experimental framework employed, the resultant findings regarding *T. micranthum* indicate the absence of cannabinoids within its leaves, inflorescences, branches, and fruits. Comparative analysis via TLC, HPLC-DAD, and GC-MS involving *T. micranthum*-derived samples vis-à-vis analytical standards of CBD, CBN, and Δ^9^-THC, alongside *C. sativa* samples demonstrated that under identical experimental conditions, cannabinoids were only detected in the *C. sativa* samples. 

Regardless of the presented outcomes, the inherent limitations in definitively proving the absence of cannabinoids in *T. micranthum* should be highlighted. For instance, the amount produced might be below the detection limits of current methods, or production could be contingent on specific environmental conditions, time of year, or even chemotypic variations within the species. However, this work provides strong evidence for the possibility of obtaining false-positive results when using less selective tests, such as colorimetric assays, or relying solely on retention times of chromatographic techniques. Thus, considering the well-known rule that the burden of proof lies with those who make the claim, new assertions that *Trema micranthum* produces cannabinoids will need the support of selective experiments.

## Data Availability

Data are contained within the article.
